# Notes and News

**Published:** 1898-09-17

**Authors:** 


### Notes and News.

(Collected and Communicated.)

We regret very much that in an advertisement, which appeared last week in our columns, announcing an examination of candidates for entry into the Medical Department of the Royal Navy, the wrong date was accidentally inserted. The examination will be held on October 31st and the following day. Fifteen commissions as surgeon will be offered for competition.

The district medical officer at Southall has instituted a bi-weekly examination of the children attending the Board schools, with a view to detecting any signs of diphtheria, which has lately been epidemic in the district. Dr. Windle, the medical officer, considers the elementary schools largely responsible for the spread of diphtheria, and hopes by means of the present experiment, which is to be continued until the end of the present year, to bring the disease under better control.

It will be a matter of great satisfaction to everyone to learn that the Hospital Saturday Fund have abandoned their street collection this year. Mr. Reginald Acland has prepared an interesting little magazine putting forth the needs of the Fund and of the charities it benefits, and besides this, on Hospital Saturday, to be held this year on October 15th, tradesmen are authorised to receive contributions to the Fund. It is to be hoped that the public, to show their appreciation of the reform, will increase the generosity of their gifts.

A bulletin has been issued by Sir William MacCormac and Mr. Alfred Fripp in regard to the condition of the Prince of Wales, in which it is stated that most satisfactory progress has been made, and that with the help of the apparatus, which it will be necessary for him to wear during the daytime for many weeks, he has already been able to walk with ease on level ground with the assistance of a single stick. Massage and passive movement are continued, and a considerable degree of mobility has been obtained in the knee joint. On Sunday morning His Royal Highness attended service in the Queen's private chapel at Osborne, and was able to walk slowly to his seat. The stay on board ship and at Osborne have been very beneficial to his general health. He goes to Scotland this week.

At the last meeting of the Metropolitan Asylums Board the question was asked whether there was any evidence of an increase of infectious disease in the area supplied by the East London Water Company? The Chairman, in reply, gave the figures, which went to show that the proportion of cases in the eight East-end parishes to the number occurring in the whole of London was last year 28 per cent., as compared with 20 per cent. this year. Ultimately a resolution was passed urging that every effort should be made to obtain an adequate supply of water at the earliest possible moment, and to prevent the recurrence of the present difficulty.

We learn that the directors of the Arbroath Infirmary have solved the dispensing difficulty, to which we referred last week, in a manner which, whether satisfactory to the present dispenser or not, will hardly commend itself to the local chemists, who, to use a legal phrase, have taken nothing by their writ. In place of Miss Kay another lady has been appointed legally qualified to dispense medicines. She is, we understand, at present acting as dispenser in a branch of the Metropolitan Provident Association, London, has excellent testimonials, and several years' experience. The choice of a lady has many solid reasons to recommend it, and we do not see why the employment of lady dispensers should not become very general, especially as there is nothing to prevent them acquiring whatever qualification may be thought necessary for filling that post. Miss Minton (Miss Kay's successor) is appointed dispener and nurse at a salary of £30 per annum and her board, an eloquent comment on the state of the skilled labour market.

The case which we referred to of ducking a doctor at the Glasgow Royal Infirmary has now been before the Courts. The result is that a fine of one guinea has been imposed upon each of those concerned in the act. The Managers of the infirmary have accepted an apology from them; and Dr. Garvie, the victim of the ducking, has tendered his resignation, which has been accepted. The case has led to a good deal of criticism of the management of the Infirmary. Much of this is beside the point. What, however, has not been cleared up and placed on a definite basis is the real use and intent of Ward XIV. Originally it seems to have been an erysipelas ward, but of late it has been used for septic cases. It is quite clear that the general opinion among the residents is that septic cases are to go to Ward XIV., and there can be no doubt that in a large institution like the Royal Infirmary, with its more than 500 beds, it is only right that such a provision should be made for patients who can only add to the risk of aseptic cases in other wards. But, according to the statement published by Dr. Garvie, "the ward has been used for ordinary surgical cases." The matter should be cleared up, and if Ward XIV. or any other ward is intended for septic and erysipelatous cases, it should be reserved for them alone, and no ordinary surgical cases should be admitted to it. Incidentally we notice that the very evil custom of using the gynæcological wards for the examination of certain of the out-patients, as well as candidates for admission, obtains at the Royal Infirmary. It is a fertile source of evil, and ought not to be allowed.

